# Inline quantitative myocardial perfusion flow mapping

**DOI:** 10.1186/1532-429X-18-S1-W8

**Published:** 2016-01-27

**Authors:** Hui Xue, Michael S Hansen, Sonia Nielles-Vallespin, Andrew E Arai, Peter Kellman

**Affiliations:** grid.94365.3d0000000122975165National Heart, Lung and Blood Institute, National Institutes of Health, Bethesda, MD USA

## Background

Quantification of myocardial blood flow (MBF) using first-pass perfusion MRI has potential to objectively evaluate ischemic heart disease. While considerable development and clinical research has been conducted during the past two decades on this topic, there lacks a perfusion flow mapping solution that runs on the MRI scanner and produces MBF maps immediately following the perfusion scan. To be effective in a clinical environment we identified following capabilities are needed: fully automated workflow, pixel-wise flow calculation, single-bolus contrast injection, complete free-breathing acquisition, rapid processing, and inline deployment.

## Methods

**Figure 1:**
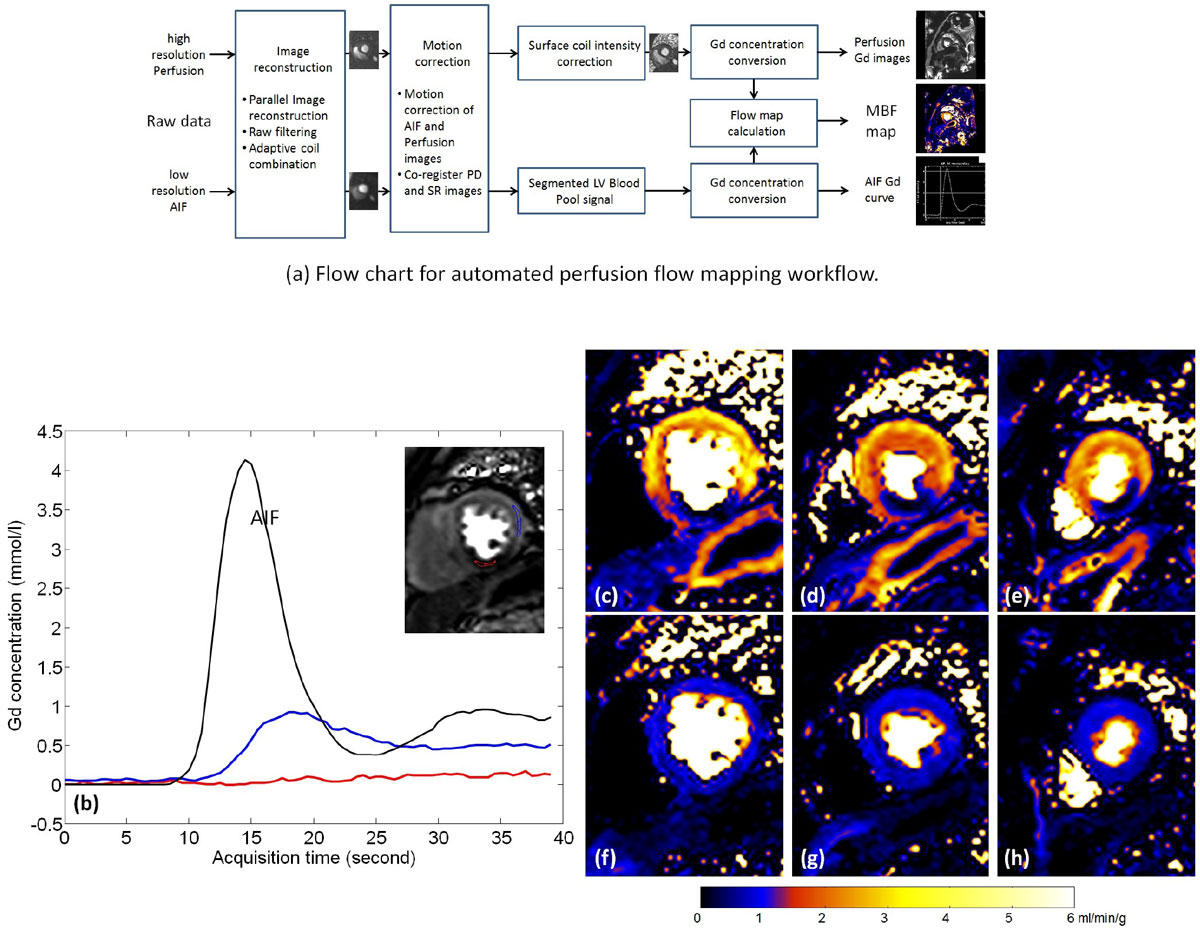
**Flow chart of proposed workflow (a) and example of inline perfusion MFB mapping**. The entire process is integrated line and flow maps are calculated without any user interaction. (b) is the Gd concentration curve for SSFP stress positive perfusion scan. For two ROIs picked in the hypoperfused (res) and normal (blue) myocardium, time Gd curves are shown. The AIF Gd curve is plotted for reference. Myocradial blood flow maps for stress and corresponding rest scane are vien in (stress, c-e; rest, f-h) which were generated using the two compartment model.

Multi-slice saturation recovery (SR) imaging was performed during the first pass of a single bolus of Gd injection with normal free breathing. A low resolution blood pool image was acquired each heart beat for the measurement of arterial input function (AIF). AIF imaging used a dual-echo FLASH sequence and correction of T2* during the first pass. Higher resolution myocardial imaging used either FLASH or SSFP protocols. Proton density (PD) weighted images were acquired prior to contrast, used for correction of surface coil intensity and conversion to Gd concentration units (mmol/L). Figure 1a illustrates the fully automated processing workflow: (1) image reconstruction, (2) motion correction (MOCO) of both AIF and perfusion images and co-registration with PD images, (3) automated segmentation of the AIF LV blood pool signal, (4) surface coil intensity correction, (5) conversion to [Gd] units from SR and PD signal intensities using Bloch equation calculations including correction of T2* loss in AIF from dual echo signal, and (6) calculation of pixel-wise MBF maps by deconvolution processing and display using a custom colormap. Three methods were implemented inline: (1) model-free method using a novel L1-norm based optimization, (2) a constrained Fermi function and (3) a two compartment model with interstitial volume estimation. The whole process was implemented in C++ via the Gadgetron framework [1] and integrated inline so that the motion corrected Gd concentration images and flow maps were calculated without any user interaction.

## Results

The pixel-wise MBF maps for all slices were created within 1.5 mins after the end of scan. Mean MBF values (Table 1) were computed from a ROI drawn in the myocardium. Rest ROIs include all myocardium. Stress positive (8 of 16 studies) ROIs was drawn remote from the hypoperfused region. The performance of motion correction and surface coil correction was visually assessed to be good to excellent for all cases. Inline MBF mapping for a stress positive study are shown in Figure 1b-h.

## Conclusions

Fully automated pixel-wise MBF mapping was integrated inline on a clinical scanner. Myocardial perfusion flow maps were computed without any user interaction. This technique could promote the clinical usage of fully quantitative perfusion flow mapping.

**Figure 2:**
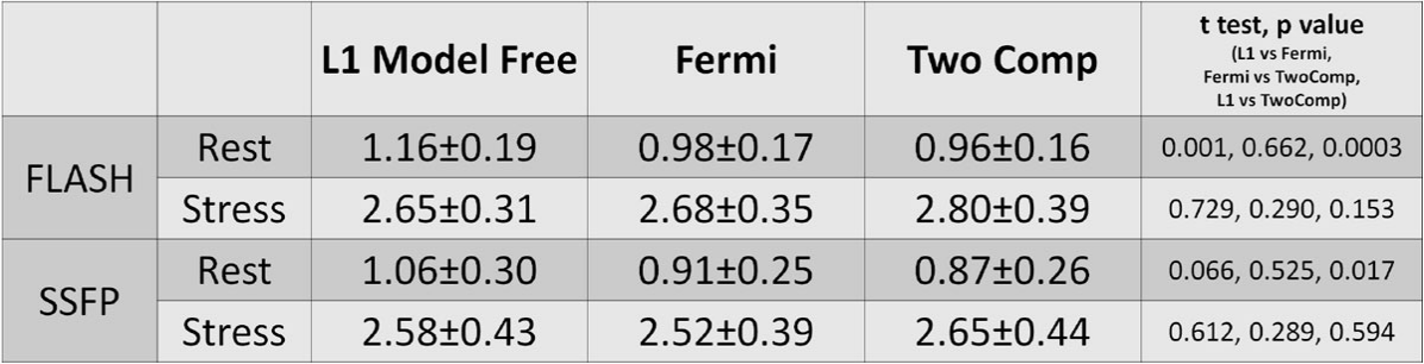
**Measured Myocardial Blood Flow values for rest and stress perfusion study**. First pass perfusion is used in L1 model free and Fermi doconvolution and the whole data ranged is used in two compartment model. N = 16 patients underwent rest and Adenosine stress perfusion studies with local IRB approval and written consent for either FLASH (N = 8) or SSFP (N = 8) protocols. Imaging experiments were performed on a 3T clinical MRI system (MAGNETOM Skyra, Siemens). 60 heart beats (including 3 PD beats) were imaged with three short-axis slices being acquired per beat. Typical imaging parameters for FLASH were: FOV 360 × 270 mm^2^, 14° flip angle, 8 mm slice thickness, interleaved parallel acceleration R = 3, acquired matrix 192 × 111, single shot imaging duration of 53 ms. SSFP protocol used 50° flip angle and had single shot duration of 67 ms. The Gd dose was 0.075 mmol/kg for FLASH and 0.05 mmol/kg for SSFP administered at 2 ml/s.

## References

[1] Hansen MS (2013). MRM.

